# Stakeholder Beliefs about Alternative Proteins: A Systematic Review

**DOI:** 10.3390/nu15040837

**Published:** 2023-02-06

**Authors:** Mario Amato, Roberta Riverso, Rossella Palmieri, Fabio Verneau, Francesco La Barbera

**Affiliations:** Department of Political Science, University of Naples Federico II, Via Rodinò 22/A, 80138 Naples, Italy

**Keywords:** sustainability, cultured meat, edible insect, plant-based, rational action approach, thematic analysis

## Abstract

In recent years, a great deal of research has been conducted on consumers’ attitudes and beliefs in favor and against alternative proteins (AP). By contrast, a much more limited research effort has been devoted to understanding stakeholders’ point of view. The current work provides a first systematic review of the existing literature on stakeholders’ beliefs towards alternative protein sources. Moreover, a secondary content analysis was carried out on the selected studies, providing an overview of the major themes emerging from the existing literature in relation to utilitarian, normative, and control beliefs that stakeholders hold towards AP. Results showed that stakeholders’ beliefs are very different from those that emerged from previous research on consumers’ views. Overall, stakeholders appear much more aware, compared to consumers, of the implications of using alternative proteins in relation to the main pillars of sustainability (economic, environmental, social). Stakeholders’ beliefs were grouped into 13 categories, such as “economics”, “consumers”, and “rules”. With respect to future scenarios, they attribute an important role to political institutions, with respect to both economic and communication aspects, which they consider crucial to overcome persistent consumer skepticism.

## 1. Introduction

The availability of sustainable diets, in particular new protein sources for both human and animal nutrition, have become of increasing interest to researchers, industry and, consequently, policy makers. Among the many contributing factors, food security and environmental protection are the most debated and analyzed by scholars, which ranked the availability of new protein sources as one of the most important food policy objectives both in the medium- and long-term [1,2,3].

Alternative protein sources present enticing opportunities for partially substituting protein sources coming from unsustainable agri-food practices (such as intensive livestock farming or overfishing), while also potentially helping to reduce the environmental and carbon footprint of agriculture, and playing a role in increasing food security; these alternative proteins include microbial proteins (microalgae and mycoproteins), insect-based proteins, cultured meat, plant-based meat substitutes, and dairy alternatives.

The alternative proteins market base is approximately around $2.2 billion [4] and it is destined to rise in the next years, reaching $290 billion by 2035 [5].

A great deal of research has investigated psychological factors that may influence the acceptance of alternative proteins and/or the proneness to meat consumption reduction [6]; in addition, efforts have been made in order to synthesize this research corpus [7,8,9]. Food neophobia—the reluctance of trying new foods—is one of the main factors that has been identified as a barrier to the acceptance of alternative protein sources [10,11,12,13,14,15,16,17]; it is a complex trait that can be influenced by a range of factors, including cultural and personal experiences, as well as individual differences in sensory and disgust sensitivity [18]. Research has also highlighted the importance of disgust, which may discourage the adoption of alternative proteins [19,20,21,22,23], and might have more explanatory power in predicting intention to eat alternative proteins when compared to food neophobia (e.g., [11], in the case of edible insects). Consumer attitudes towards alternative proteins are also influenced by a range of factors, including personal values, dietary preferences, and the perceived environmental and health impacts of these products. In addition, younger consumers and those who identify as vegetarian, or vegan may be more open to trying alternative proteins [22,24,25]. On the other hand, there are also factors that may discourage the adoption of alternative proteins, such as a lack of availability or concerns about the taste and texture of these products [26].

This paramount research has been almost totally devoted to the understanding of consumers’ beliefs about AP, whereas there is a substantial lack of research regarding stakeholders’ beliefs. Stakeholders are intended here as groups or individuals who can affect or are affected by the achievement of specific economic objectives [27]. Stakeholders are groups or individuals on which the market sector depends for its success. These groups may include employees, suppliers, and shareholders, but also public groups, such as governments and communities that provide infrastructure and indirectly regulate the market activities [28]. The lack of consideration devoted to stakeholders’ attitudes and beliefs is somewhat surprising, because their role is essential to determine where the supply chains will be directed and how the market will respond. Furthermore, no effort has been made so far to synthetize the existing studies that addressed the issue of stakeholders’ beliefs towards alternative proteins. In the current paper, we provide a first review and a secondary analysis of research conducted so far with stakeholders in the AP sector.

### Aims of the Study

As noted above, research on alternative proteins has focused on consumer attitudes as well as on nutritional values of alternative proteins, whilst less has been conducted with regards to stakeholders’ beliefs in relation to AP, and their beliefs about consumers’ attitude towards AP as well. In the current paper, we provided a first systematic review of the existing literature. After that, we conducted a secondary analysis of the results that emerged from the studies included in the review, to synthesize the modal sets of stakeholders’ beliefs and the main themes emerging from the literature reviewed.

## 2. Materials and Methods

The literature review was conducted and reported in accordance with the PRISMA 2020 statement [29] and was not pre-registered. The literature search was conducted on Scopus and Google Scholar, with no limit regarding subject, discipline, and year of publication. Ten keywords were used for the search, divided in two categories. The first category was related to studies’ participants (stakeholder, producer, key-people), the second was related to AP (alternative proteins, insect, algae, cultured meat, cellular agriculture, in vitro meat, plant-based). Several rounds of research were carried out with all the possible combinations between the terms in the two categories, connected by the operator “AND”: stakeholder AND alternative proteins; producer AND algae; key-people AND cultured meat, and so forth. In this research, conducted between September and October 2022, 71 articles were identified. Subsequently, these articles were further selected, using several methodological criteria. We excluded: (1) articles not relevant to the subject of the search (21 items excluded); (2) articles that did not contain empirical research directly involving stakeholders (e.g., by means of interviews, focus groups, and so forth) (18 items excluded); and (3) conference materials, dissertations, and book chapters (10 items excluded). The 23 remaining articles constitute the final corpus and satisfy the following inclusion criteria: (1) empirical studies with stakeholders as participants; (2) articles published in peer-reviewed journals in English; and (3) studies evaluating alternative proteins (e.g., algae, insects, cultured meat) as meat substitutes. The selected papers cover roughly a decade, from 2013 to 2022. Among the 23 items analyzed, five articles explored alternative proteins in general and one article addressed plant-based food products. A substantial number of studies (11) focused on cultured meat, whereas in two research, both in vitro meat and plant-based products were considered. Three articles sought to investigate stakeholders’ beliefs regarding the production of algae-based foods and only one article was on insects. The full list of selected papers is provided in Table 1.

## 3. Review of Selected Papers

Research on alternative proteins (AP) began about a decade ago in the California Silicon Valley region, thanks to funding from large corporations. Since then, the region has become a reference point for many other companies in the food sector. Sexton [34] analyzed the cultural, social, and economic factors underlying the development of AP. Through forty-one semi-structured interviews conducted with key stakeholders working in the commercial development of cellular agriculture and plant-based products, it emerged that many of the respondents chose to voluntarily relocate to Silicon Valley to start their businesses, because the region attracts a concentration of public and private institutions and human resources that can ensure the success of innovative products. 

Tziva and colleagues [35] explored the dynamics of innovation linked to the supply chain of foods produced with alternative proteins. Through a chronological review of the events and thirty in-depth interviews with stakeholders belonging to the non-meat food chain—representatives of the political world and NGO—it emerged that since the 1990s, vegetarian and vegan consumers have pushed a small number of companies to experiment with new food products. For many years, these products have been part of a niche market. However, this market, which was initially restricted, has begun to expand, bringing profit to the manufacturing companies. Subsequently, driven by these new trends, other companies began to move in the same direction. The need to replace meat has subsequently taken on a connotation that is not only ethical, but also eco-sustainable. Therefore, according to these stakeholders, the pressure towards the protein transition exerted by minority social groups was welcomed and eventually promoted by political and institutional actors.

In one of the first studies conducted on cultured meat and stakeholders, Chiles [37] explored the perceived consequences of introducing novel foods to the market. From this pioneering research, it emerged that stakeholders were rather skeptical about the success of these novel foods: first, for cultural reasons, because cultured meat may not be perceived by consumers as “real meat”; and second, because to produce cultured meat, there is a need of extremely advanced technological support, which was lacking in those years. Similar elements emerged from another research conducted by Chiles [38]: stakeholders in the scientific world believe that in vitro meat is a valid alternative to conventional meat, as its production is respectful of the environment and animal welfare; however, an obstacle to its diffusion may be the reaction of consumers, who will have to be guided by good communication.

As regards algae, Moons et al. [14] conducted a multi-stakeholder qualitative study aiming to outline the early adopter market segments for Spirulina-enhanced foods. Three target segments emerged: “sporting individuals”, “vegetarians”, and “foodies”. These three segments would be interested in the consumption of foods containing AP for health, environmental, and ethical reasons, regardless of how sensorially attractive these foods are. In 2015, Vigani and colleagues [31] studied the production, distribution, costs, and extraction methods of algae, used as food. According to the stakeholders interviewed, one of the factors that negatively affects the spread of microalgae in Europe is the low demand from consumers who do not show interest in these novel foods, as they have little knowledge about them. In the opinion of the experts interviewed, it is possible to increase demand by introducing specific nutrition education programs.

Bohm and colleagues [36] also reached similar conclusions and, in a research conducted with 12 German stakeholders, underline the importance of good communication that can persuade consumers about the benefits of alternative proteins. Specifically, the interviews involved scientific experts in the cultured meat sector. They pointed out that the real problem is that cultured meat is perceived by consumers as a “non-natural” product and this perception could negatively affect the purchase choice. However, this negative attitude of consumers, according to the respondents, could easily be overcome by the awareness of three factors that have large benefits: (1) cultured meat responds to environmental issues, since it produces a lower emission of greenhouse gases than conventional meat; (2) responds to ethical issues, as it foresees a significant decrease in animal exploitation; (3) and encourages ecological agriculture, reducing intensive methods. However, for consumers to be convinced of the benefits of this new food, good communication is needed.

The importance of communication and discursive practices is the focus of Sexton’s research [33], which analyzed the biopolitics of consumption. This study, conducted through 25 semi-structured interviews conducted on founders, employees, and investors of private companies operating in the AP sector, located in California, highlighted the importance assumed by discursive practices as a normalization tool that leads to the acceptance of these new food products. In fact, providing new information allows consumers to restructure cognitive patterns related to food edibility. With the same interest for communication, the study conducted in 2019 by Sexton and colleagues [1] examined how manufacturers and retailers “tell” consumers about food products that replace meat, milk, and all other foods of conventional animal origin. The research was conducted through 30 semi-structured interviews with company founders, employees, investors, third-sector advocacy groups, and other key stakeholders. They found that, to create consumer demand, AP narratives have so far been characterized by several “promises”: (1) “Healthier bodies”; (2) “Feeding the world, now and forever”; (3) “Good for animals and the environment”; (4) “Control for sale”; and (5) “Tastes like animal”. The narratives of stakeholders, therefore, try to portray AP-based foods as perfectly balanced between clean eating and good for health, without guilt towards animals and the environment; and with a taste similar to traditional products.

The importance of these aspects also emerged from the 12 interviews conducted by Woll and Bohm [46] with stakeholders belonging to environmental associations. When talking about cultured meat as an alternative protein, respondents believe that it holds the promise of the future, as it is environmentally sustainable, ensures animal protection, but, above all, reduces cardiovascular diseases and hypertension. However, the same interviews show that, as important as the environmental issue is, respondents are aware that consumer choices will be oriented by taste and flavor. As one of the respondents stated: “… an absolutely necessary condition is convincing consumers in terms of taste”. Taste and appearance are potential barriers that also emerged from the study by Specht and colleagues [50], who conducted 19 interviews with German stakeholders to study the beliefs they have about insects and algae as new protein-rich foods. Respondents emphasize the importance of a negative consumer attitude, as the taste and appearance of these food products evoke disgust. Similarly, Herrick and colleagues [40] showed that stakeholders acknowledge the AP’s benefits both for the environment (as their production requires less energy) and for health (as they do not contain hormones and antibiotics); in addition, AP are ethical because they do not require animal abuse. Nevertheless, what it is really important for consumers is taste, together with more affordability.

Similar beliefs also emerged as significant from interviews with Scandinavian stakeholders [32], who highlighted aspects that could determine the success/failure of the protein transition. Food quality, guarantee of environment and health protection, respect for the socio-cultural characteristics of the population, and production costs are identified as major factors. The quality of food is, perhaps, the most important, because it determines consumer acceptance and refers to taste, aesthetics, perception of authenticity, and territoriality.

Blanco-Gutiérrez and collaborators [49] explored the beliefs of stakeholders inside and outside the Spanish agri-food chain. Participants were asked to indicate factors they believed may determine the success of AP foods. Six criteria were identified: (1) economic, (2) social, (3) environmental, (4) political, (5) culinary characteristics, and (6) technological. The results show that, according to stakeholders, the most important are the economic factors—understood as the accessibility of products—followed by culinary characteristics. The economic factor can be a deterrent to the large-scale spread of AP-based foods, and these foods risk to constitute a niche market, reserved for a few people. This is the belief that also emerged from Newton and Blaustein-Rejto [48] interviews with stakeholders chosen from representatives of cultured meat companies; plant-based meat companies; non-profit organizations; funding agencies; government agencies and beef, soybean, and pea sectors; and farmers. Stakeholders stress the importance of food prices being accessible to the entire population, to avoid creating a niche market for narrow elites. Although respondents acknowledge that the market for alternative proteins and cultured meat has increased in recent years, and is growing further, they do not believe that it can entirely replace the animal meat market. In addition, many respondents are concerned about the social consequences of this change, especially for livestock farmers, who should diversify their production by switching to the cultivation of plants, micro-protein algae, algae, or other alternative protein products.

Serious concerns also emerge from the analysis conducted by Rumin and colleagues [30], who explored, through the administration of an online survey, the beliefs of stakeholders regarding the challenges and opportunities related to the algae food market. The survey involved stakeholders mainly from France, Spain, Portugal, and the United Kingdom. The main limitations to the spread of microalgae, according to respondents, are of an economic nature; they are linked to production costs and technologies, but also to the cost of approval of the marketing of microalgae-based products that meet legislative requirements. Legislation appears to be another major limiting element: the most part of the stakeholders interviewed highlighted the absence of legislative competences related to the sector. In fact, stakeholders complain about the difficulties in obtaining authorizations to produce new foods.

Brazilian stakeholders who participated in the study by Reis and colleagues [44] have placed the issue of social justice at the center of attention. Respondents pointed out that low-income countries need a higher protein diet, which could be guaranteed through large-scale cultured meat production. However, respondents also underlined that the high costs and lack of sensory appeal of cultured meat may represent a significant limitation. Morais-da-Silva and colleagues [47] also conducted 35 semi-structured interviews with Brazilian stakeholders of meat and alternative products companies, to explore the risks and opportunities that experts see in the transition from a meat-based market to a plant-based meat production. Most stakeholders cited among the opportunities of restructuring the production chain, with the creation of new jobs, especially in the management, marketing, and advertising sectors. Respondents also believe that for these new products to establish on the market, it is necessary that prices make them accessible to the entire population, including those with a low to middle income. 

The stakeholders interviewed in China by Dempsey and Bryant [39] seem optimistic: Chinese consumers are extremely sensitive to environmental issues and the Chinese government is very interested in food safety, given the population density and the number of inhabitants. According to respondents, these factors could have a positive effect on the spread of cultured meat in the country, which could be introduced to the market within a few years. Negative beliefs were expressed instead by Finnish stakeholders surveyed by McPartlin [42]. According to respondents, the introduction of cellular agriculture could generate a radical change in rural areas. The main fear that emerged is about the risk of production of cultured meat to be concentrated in the hands of large companies, as they have enough capital to invest in this innovative sector, endangering the survival of small farms. Moreover, according to the interviewees, since this is a new market, European legislation regulating all aspects should be needed. The need for global legislation regulating cultured meat production also emerged from the interviews conducted by Ketelings et al. [41]: the existence of uneven legislation at a global level could hinder the placing on the market of these novel foods, which are not well known; therefore, may be considered as risky for health. For this reason, respondents believe that it is necessary to ensure safety and transparency throughout the production chain. The theme of transparency is also relevant in the study by Moritz et al. [43]. One of the interviewees claims that: “It is important to have strict food controls, produce clear guidelines as to what may be added in such products and to exercise a high degree of transparency so that the consumers can evaluate what they are eating.” Respondents acknowledge that novel foods could meet environmental needs and food security, yet are concerned that this production could undermine the agricultural system and the biodiversity of pastures in Germany, as farm animals may no longer be needed. In addition, widespread among respondents is the fear of the emergence of a monopoly or a productive oligopoly held by a few large companies. One respondent stated: “Whenever a monopoly is created, we have to deal with massive consequential problems. […] In the face of the 21st century, I believe that we have to work more with open source and that the knowledge should be shared by many brands”. Similar fear emerged from interviews conducted in Australia in a previous study by Sievert and colleagues [45]: stakeholders fear that production power will pass into the hands of large companies, which seek to increase their profits by investing in this new food sector.

## 4. A Secondary Analysis of Stakeholders’ Beliefs

A secondary content analysis has been conducted on the stakeholders’ beliefs reported in the same research corpus reviewed in the previous section. The analysis—carried out with the support of the software ATLAS.ti—aimed to synthesize the rich range of opinions and expectations that emerged from the reviewed studies in terms of the respondents’ beliefs. A thematic analysis of the qualitative data retrieved from the studies was conducted, with a progressive process of abstraction in which the single quotes were grouped into categories; then, grouped into main themes; and finally, linked together when appropriate. The analysis has been conducted by three researchers—who are among the authors of the current article—with different expertise (economics, social psychology, and human nutrition), who have independently analyzed the texts; final decisions and interpretations have been based on the consensus of all three [51,52]. The thematic analysis, a qualitative content analysis method, does not involve statistical inferences. ATLAS.it was used only in support of the analysis, which was made by researchers through the interpretation and categorization of textual materials in a hermeneutic epistemological framework.

Drawing on the rational action approach [53], we distinguished between three categories of beliefs: (1) expectations about positive/negative consequences of the introduction/development of AP (utilitarian beliefs); (2) beliefs about others’ (individuals or groups) attitudes and behaviors (normative beliefs) inherent to AP; and (3) beliefs about facilitating and hindering factors (control beliefs) relevant to the AP market [52]. We analyzed and grouped beliefs with reference to this threefold grid, as follows.

### 4.1. Utilitarian Beliefs

First, we analyzed the beliefs about positive/negative consequences of the introduction of alternative proteins into the market and/or individuals’ diet. In this area, a first category of beliefs makes explicit reference to positive and negative beliefs about environmental consequences of introducing AP (category: ENVIRONMENT). Overall, most of the opinions collected by the studies in our literature review are positively connotated [36,45,48]. The positive expectations about consequences of the AP introduction are mostly related to a decrease in environmental exploitation (“It consumes less water, fewer resources and emits less greenhouse gases.” [39]), which is strictly correlated to economic considerations about the efficiency of AP [40,50] that we further discuss below. Nevertheless, there are also negative beliefs about environmental consequences of AP, as regards the changes imposed to traditional agriculture and landscapes [36,42]: “the agricultural or traditional landscape and grassland biodiversity would be negatively impacted if livestock animals were no longer needed” [43].

A second category of beliefs regards the economic area (category: ECONOMICS), which groups positive and negative beliefs about economic consequences of introducing AP. Into this category, a major subgroup of beliefs refers to the efficiency of AP, as already described above, in terms of reducing the exploitation of resources. In addition, stakeholders also notice that AP may have an extended shelf life and may not require cold storage [40]. Other expected advantages are the opportunity to diversify production for large producers, the possibility that new sectors may emerge, the re-vitalization of agriculture, and the creation of new job opportunities, also in rural areas [36,47,48]. The study by Morais-da-Silva and colleagues also provides many expressions of positive expectations about job improvement, in terms of opportunity, salaries, and fairness: “I think there will be jobs for every-one. We will create more jobs across the entire new chain”; “Every technology and every innovation always bring with them an increase in the quality of labor”; “Working in an alternative meat factory will be much better than working in a slaughterhouse” [47].

One major negative belief expressed by several stakeholders refers to the possibility of monopolization or “oligarchy” of this new market perpetrated by large companies at the expense of the small ones. This could be due to the need of large investment in technologies and research, especially in the early moments of market creation and development; and specially with reference to cultured meat, which seems to be perceived as the most demanding in terms of economic investments [42,43,45,48]. Nevertheless, according to other stakeholders, even if larger producers might meet the needs of this new market more quickly due to their financial and technological resources, this market opportunity may also be extended to small farms through inclusive policies and cooperative initiatives [47]. 

A third category groups the beliefs that make explicit reference to animal welfare (category: ANIMAL WELFARE). This is one area in which only positive beliefs did emerge [38,39,44,45]. According to the interviewed stakeholders, the introduction of AP “could help to achieve a future world without the exploitation of animals” [36], and avoids animals’ sufferings [40,47]. Noticeably, beliefs about positive expected consequences on animal welfare are expressed by most of the stakeholders in the cultured meat sector, whereas there is no evidence of the presence of this kind of beliefs among stakeholders involved in sectors based on algae and insects.

Ambivalent beliefs are instead reported in relation to AP and human health (category: HEALTH). For example, cultured meat—which is often represented or evaluated in comparison with traditional meat—is portrayed by stakeholders as a healthier option compared to the traditional one; nevertheless, people may be pushed to an excess meat consumption because it becomes ethically correct [36]. Overall, in vitro meat scientists and producers portrayed cultured meat as a healthy and safe food product [1,37,45,48,49] because it may have a high nutritional value, yet it would not contain hormones, antibiotics, or bacteria of animal origin [39,40]. Cultured meat could be safer and healthier because the nutritional profile can be pre-determined: “Cultivated meat is openly engineered and you may include more or less sodium, vitamin D, calcium, whatever you want for people’s health” [47], so that it may foster a reduction of cardiovascular diseases, obesity, hypertension, and diabetes [46]. Nevertheless, other key people believe that the safety of the product is still unknown [41]. Hence, in the health domain, there is a polarization of beliefs about cultured meat, which is perceived as a safe, but also as a risky object. Interestingly, neither a special concern about health—nor beliefs about positive consequences—seem to emerge as regards algae and insects.

One last important category of expected consequences refers to FOOD SECURITY; that is, the availability of nutritious and safe food. As regards this category, from the analysis of the selected study, it is easy to notice that positive expectations about the introduction of AP, in terms of food security, are widespread among stakeholders [39,47]. First, AP are expected to increase significantly the overall amount of available proteins [43]; thus, they could help solve the challenge of accessing proteins even in low-income countries [32,47] and for specific categories of people (e.g., rural residents—[48]).

### 4.2. Normative Beliefs

In line with the theoretical framework that guides the review, a second class of beliefs that we analyzed groups together the opinions and statements that stakeholders produced in relation to the perceived normative pressure in favor or against the development and widening of the AP sector. In other terms, we selected beliefs—reported in the reviewed studies—that referred to individuals, groups, and entities that are thought to be (or to exert social influence) in favor or against AP. At the macro-social level, we found explicit references to the national governments and political institutions, which provide and guide the policies concerning AP (category: INSTITUTIONS). According to the respondents, institutions are interested in and support AP mostly as a function of their concern about food safety and food security, especially in the most populated countries (such as China) and in emerging economies [32,35,39,44]. At the individual level (category: CONSUMERS), stakeholders acknowledge that the AP market is strongly dependent on consumers’ demand, attitude, and desire; and consumers are influenced by their beliefs about the preferences and behaviors of others [40,43]. At the intermediate level between individuals and institutions, different social groups are considered by respondents as relevant in influencing the AP market (SOCIAL GROUPS). The main motives that characterize these groups concern the environment, sustainability, and animal welfare; thus, these groups are thought to exert a significant pressure in favor of the AP market [35,39]. They are differently described as environmentalists, vegan, and vegetarians; some respondents also refer to elite groups of policymakers and scientists, which are supposed to exert a deep impact on AP: “The emerging awareness of sustainability within a small group of policymakers and scientists has led to expectations for the environmental improvement potential of the wider uptake of meat substitutes.” [35]. Finally, there are respondents who underline the pressure exerted by large companies: “Big companies […] pretend to promote environmental protection but promote their profits promoting the market for new proteins” [45].

### 4.3. Control Beliefs

In recent developments of the rational action approach, together with beliefs concerning consequences and social pressure, a third order of considerations refer to the factors that individuals believe may facilitate or hinder the phenomenon of interest. Therefore, a final class of beliefs regards the respondents’ expectations about which factors may foster or jeopardize the AP market. A major set of beliefs stakeholders express is related to the economic domain (category: PRICES&COSTS). These beliefs are different when referred to producers or consumers. In relation to the production domain, stakeholders express a major concern about the costs of AP, which are expected to be higher compared to those of conventional proteins; thus, hindering the development of this novel market. The high costs, especially in the early phases of the large-scale production and commercialization, together with the competition with mature markets (crops, land plants) are expected to be a major obstacle and to foster the risk of monopolization of the market by large companies [30,40,50]. High production volumes and a dramatic reduction of production costs are thought as necessary to be competitive with agricultural commodities [31]. A similar and specular concern is expressed by stakeholders thinking about the consumers’ perspective, in relation to which price becomes a key factor in promoting this market [43,49]. Consumers are defined as very “price-sensitive”, so that they would not buy AP-based food if it were too expensive [36]. However, several experts are more optimistic, and believe that investments in research and improvements of the production processes may help reduce the prices for consumers, which will tend to decrease in the near future [44,47].

Another cluster of beliefs inherent to consumers’ acceptance is related to AP’s sensory characteristics (category: SENSORY). According to stakeholders’, taste, appearance, and flavor are key elements for consumers’ acceptance of AP, even more important than environmental or health issues [36,40,44,49]. For the AP market to expand, a necessary condition is convincing consumers in terms of taste [46]. Strategies to make AP familiar and appealing are needed to engage consumers [33]. 

Another concern stakeholders express in relation to consumers regards their SKEPTICISM, which is thought to be rooted in culture and tradition: “If you’re going to celebrate your wedding anniversary, you’re going to buy a mature steak from a young calf” [47]. They seem doubtful that consumers will ever overcome this cultural skepticism [38]. This is strictly linked to another limiting factor, namely the perceived lack of naturalness (category: NATURALNESS) of alternative proteins. As noted by one respondent from Woll and Böhm’s research [46], “Eating is one of the most natural things in the world. The trend goes first towards naturalness”. Cultured meat, as already noticed, is often evaluated in comparison to traditional meat; in this framework, negative beliefs seem to emerge in relation to this kind of food: “products that are labeled as ‘meat’ should be limited to those that are derived from the tissue or flesh of an animal harvested in the traditional manner” [1]. The production of in vitro meat is not seen as a natural process [36,38].

A final major concern expressed by stakeholders refers to the (lack of) legislation in the AP sector (category: RULES). According to stakeholders, there is a lack of legislation at global level that prevents, for example, the development of a cultured meat market. The situation in EU seems to be better compared to other countries (such as China), and the current legislation might be sufficient to guarantee the introduction of safe products on the market [39,41,42]; nevertheless, in the sector of algae, stakeholders complain of a too strict legal framework in the EU [31]. Regulatory clarity is fundamental to provide a clear path to market products based on alternative proteins [48]. Instead, according to several stakeholders, the legal situation is unclear or complex, which prevents operators from developing their business activities [50].

As regards major factors that may foster the AP sector, most stakeholders indicate communication as a key element (category: COMMUNICATION) [36,44]. Information on AP needs to be communicated clearly to consumers, in order to overcome skepticism and promote a positive view of these novel foods:

“The process of producing cultured meat is largely unknown. Information on this and the reasons for it should be communicated to consumers. Especially in the development phase, when companies and researchers are in the process of organizing and starting the production process, it is important to involve consumers to close information gaps or to reduce prejudices”.

[43]

**Figure 1:**
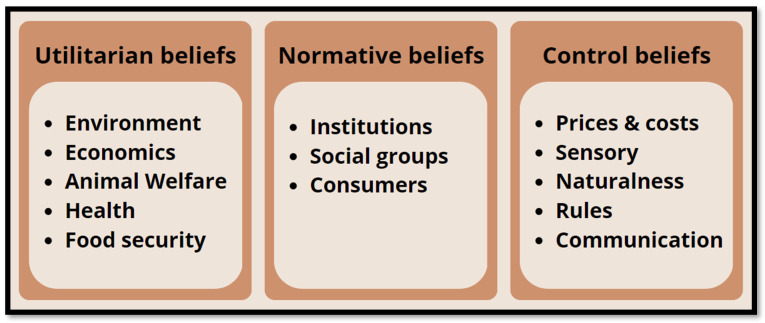
Main categories of beliefs.

The topic of communication meets the topic of rules when stakeholders express their beliefs on the importance of transparency, certification, and traceability of the AP-based food products, which are felt as other elements with a relevant potential of exerting a positive influence on consumers [43,46]. The themes of communication, rules, and transparency, in the stakeholders’ view, call into action governments and institutions, which have a primary role in the developing of the AP market. Institutional support in relation to communication, legislation, transparency, and also research and early investments can make a difference in the future of the AP field [14,33,39,47,48]. An overview of the main categories of beliefs emerged is showed in Figure 1.

## 5. Discussion

The food market is in transition, as the current system is not environmentally sustainable. In recent years, studies have multiplied that have tried to analyze the obstacles and concerns that this market will encounter. Most research has focused on consumers’ attitudes and beliefs towards alternative proteins. Overall, while scientists steadily underline the benefits of AP, research shows that consumers are not aware of the environmental and nutritional benefits of consuming alternative proteins (if not provided with specific information during the studies; see, e.g., [54]), and almost ignore the environmental impact of meat production. The beliefs and attitudes that consumers mostly express when surveyed are into the area of disgust/dislike, as well as in the correlated area of tradition/novelty and sensory aspects (taste, texture, appearance, and so forth) [8,14,55].

The current work shows that stakeholders’ beliefs are very different from those that emerged from research on consumers. Overall, stakeholders appear much more aware of the social, economic, and environmental consequences of a dietary conversion to alternative proteins. The beliefs they express are rich and articulated around conceptual cores that largely correspond to the main pillars of sustainability (economic, environmental, social). Among the APs considered in the present work, the cultured meat sector is still the one far removed from the market dimension. It is not surprising that cultured meats are almost absent in the authorization applications submitted by companies to the EFSA for novel foods, unlike insects or plant-based foods, where companies are already equipped to produce specific food products [56]. The placement of cultured meat at a very early stage in the life cycle of the product category justifies the extensive ongoing debate among stakeholders and is reflected in the present review in a higher number of scientific papers compared with the other sectors of insects, algae, and plant-based proteins.

The critical analysis of the present review clearly shows that the economics of alternative proteins is one of the main areas of debate among stakeholders. Overall, stakeholders’ beliefs have shown that the economics of alternative proteins is complex and multifaceted. Among the major positive expectations, stakeholders mentioned the environmental and ethical benefits of alternative proteins. These benefits in terms of reduced pressure on the environment and increased animal welfare may justify the higher production cost, particularly if externalities such as the environmental impacts of the livestock sector are taken into account. The adoption of alternative proteins could also lead to an expansion of the market and to the development of new industries, potentially leading to economic benefits in the long term.

Conversely, several areas of concern linked to the economic aspects emerged. First, the production of alternative proteins is currently considered more expensive compared to traditional protein sources, which may limit their adoption. High production costs could determine higher prices for consumers. This is particularly true for lab-grown meat, which requires innovative technologies and R&D investment. However, as production processes and technologies improve, it is expected that the cost of producing alternative proteins will decrease, making them more competitive with traditional proteins. A further frequently recalled theme is related to the potential consequences of the market power and the oligopolistic organization of the emerging alternative protein industry with negative implications for competition, innovation, and consumer welfare. High levels of market concentration may lead to higher prices and reduced competition, while lower levels of concentration may promote a more competitive and dynamic market. It is therefore important to monitor and regulate market power and concentration in the alternative protein sector to ensure that it promotes economic efficiency and consumer welfare. Finally, again on the economic side, the issue of equity and social justice emerges. Some stakeholders have pointed out that in low-income countries where it is more beneficial to ensure protein-rich diets, higher consumer prices could severely limit economic access; thus, undermining one of the basic pillars of food security.

The environmental pillar of sustainability also frequently recurs in the opinions expressed by stakeholders, even though environmental aspects are often assessed alongside economic and or social ones. For instance, the potential benefits of AP in terms of reduced pressure on the environment and increased animal welfare are considered factors that can justify and counterbalance the higher production cost, particularly if externalities such as the environmental impacts of the livestock sector are considered. Even if the social pillar is never explicitly mentioned, several factors linked to this pillar are considered by stakeholders. Potential new job opportunities, especially in marginal rural areas and developing countries, is a major qualifying aspect falling under the social pillar of sustainability. Potential impacts on human health, animal welfare, and other ethical concerns are aspects mentioned by stakeholders and which relate to the social pillar.

With respect to future scenarios, stakeholders attribute an important role to political institutions with respect to different issues. First, from an economic point of view, governments are seen as powerful actors, because their intervention might support early phases of the market development, which imply high costs, and investments in research and technologies. The intervention of governments and political institutions might be a mitigating factor as regards the risk of an oligopoly of large companies already cited, which is one of the main concerns of stakeholders. Second, institutions should have a key role in regulating the AP supply chain, with clear rules and procedure, which stakeholders largely feel to be insufficient at present. Third, institutions are called into action in relation to communication and education policies, which are considered as crucial to overcome the persistent consumer skepticism against AP.

## 6. Limitations of the Study

The study presents several limitations that need to be acknowledged. Generally speaking, the review and secondary analysis of a qualitative research corpus neither aim to—nor consent—to the generalization of results. In addition to this general consideration, the reader may take into account several points in considering the study results. First, as we already noticed, the study of stakeholders’ beliefs on AP has not been characterized by a scholarly effort comparable to the study of consumers’ beliefs. In addition, in order to reach its aim, the current review was characterized by inclusion and exclusion criteria—described in the Materials and Methods section—such as the presence of an empirical study of stakeholder beliefs in the article to be selected. Therefore, the research corpus reviewed is limited to 23 articles. Although the number of articles reviewed cannot be interpreted as a limitation per se, the reader should be aware that the considerations and interpretations provided in the study are based on a somewhat small research corpus. In addition, the research corpus did not equally represent the different kinds of alternative proteins, because most studies are on cultured meat and only three studies regard less-developed countries. The overall caution about the generalization of results should, thus, be reinforced. Second, several keywords were used for the literature search. In both categories—participants and proteins—we used words referring to the most general and wide concepts, such as “stakeholders”, “key-people”, and “alternative protein”. Nevertheless, neither all the possible kinds of alternative proteins were addressed, nor all possible categories of stakeholders. From a preliminary screening conducted, the addition of further—more specific and narrow—keywords, such as “employees” and “suppliers”, would not provide additional articles to review. This is reasonable because narrower keywords may be already covered by broader keywords. Nevertheless, there is nothing preventing further research to expand the number and connotations of keywords, and to use different inclusion and exclusion criteria, aiming to a wider research corpus to review.

As a final remark, it has to be acknowledged that the research corpus which was reviewed in the current paper was exclusively in English. This was among the inclusion criteria set for the review, as explained in the Materials and Methods section. Nevertheless, it would be interesting to conduct a more inclusive review with languages other than English. 

## 7. Conclusions

The review had the aim to investigate through the conscious gaze of stakeholders the beliefs on a new food market, in an emerging phase. A broad picture emerged that reflects the complexity of an ongoing transition in which the interests and opportunities of the various counterparts intersect. It was possible to offer such a broad overview because stakeholders, in making business choices, mediate between the requests coming from the political system; the market demand coming from consumers; and the human, economic, technological, and productive resources of the companies that are part of the entire production chain. If research conducted on consumers shows that their attitudes and behaviours are mainly driven by eating habits, taste, and economic considerations, the beliefs of stakeholders are instead characterized by a clear awareness of factors and consequences linked to AP, which are inherent to the three pillars of sustainability. Future scenarios for the adoption of alternative proteins depend on the actions of political institutions, research and development efforts, and consumer education and awareness. In order to facilitate the shift towards alternative proteins, it will be important to address the concerns identified by stakeholders, and to find ways to make alternative proteins more cost-effective and appealing to consumers. This could include efforts to improve production processes and technologies, as well as efforts to educate consumers about the benefits of alternative proteins and to create a more supportive regulatory environment.

## Figures and Tables

**Table 1:** List of papers reviewed.

Topic	References	Country	Participants	Methodology
Algae	Moons et al. [14]	Belgium	Experts in novel food markets, food companies, and health consultants (N = 10)	Workshop
	Rumin et al. [30]	France, Spain, Portugal, UK	Scientists, private companies, non-profit institute (N = 53)	Online survey
	Vigani et al. [31]	UE	Experts in the agri-food sector, scientists (N = 229)	Survey and interviews
Alternative proteins	Paloviita [32]	Finland	Scientists, processors, policymakers (N = 28)	Focus and interviews
	Sexton [33]	USA	Founders, employees, and investors of private companies (N = 25)	Semi-structured interviews
	Sexton et al. [1]	Europe and USA	Representative of start-up and non-profit organisations (N = 12)	Semi-structured interviews
	Sexton [34]	USA	Entrepreneurs (N = 41)	Semi-structured interviews
	Tziva et al. [35]	The Netherlands	Policymakers, scientists, and NGOs members (N = 30)	Semi-structured interviews
Cultured meat	Bohm et al. [36]	Germany	Scientists and entrepreneurs (N = 12)	Semi-structured interviews
	Chiles [37]	USA	Scientists, environmentalists, retailers, policymakers, animal advocates (N = 22)	Telephone interviews
	Chiles [38]	USA	Scientists, environmentalists, retailers, policymakers, animal advocates (N = 22)	Semi-structured interviews
	Dempsey & Bryant [39]	China	Industry, investors, start-ups, and third sector representatives (N = 17)	Interviews
	Herrick et al. [40]	USA	Processors, food aid, and other non-profit organization members (N = 25)	Interviews
	Ketelings et al. [41]	UE	Legislation experts, scientists, industry representatives (N = 15)	Semi-structured interviews
	McPartlin [42]	Finland	Policymakers, NGOs, food tech, and research (N = 15)	Face-to-face interviews
	Moritz et al. [43]	Germany	Policymakers, environmental and animal welfare, NGO, scientists (N = 13)	Semi-structured interviews
	Reis et al. [44]	Brazil and USA	Top managers, scientists (N = 175)	Web analysis
	Sievert et al. [45]	Australia	Food system representatives (N = 32)	Semi-structured interviews
	Woll & Bohm [46]	Germany	Policymakers, animal advocates (N = 12)	Semi-structured interviews
Cultured and plant-based meat	Morais-da-Silva et al. [47]	Brazil	Policymakers, private sector, scientists, non-profit organizations (N = 35)	Semi-structured interviews
	Newton & Blaustein-Rejto [48]	USA	AP companies, NGOs, government agencies, scientists, and farmers (N = 37)	Semi-structured interviews
Plant-based meat	Blanco-Gutierrez et al. [49]	Spain	Producers, food retailers, policymakers, scientists, and environmentalists (N = 63)	Face-to-face interviews
Insects	Specht et al. [50]	GER and USA	Scientists (N = 19)	Qualitative interviews

## Data Availability

Not applicable.
